# Among the shapeshifters: parasite-induced morphologies in ants (Hymenoptera, Formicidae) and their relevance within the EcoEvoDevo framework

**DOI:** 10.1186/s13227-021-00173-2

**Published:** 2021-03-02

**Authors:** Alice Laciny

**Affiliations:** Konrad Lorenz Institute for Evolution and Cognition Research, Martinstraße 12, 3400 Klosterneuburg, Austria

**Keywords:** Review, Social insects, Parasitology, Morphology, Nematoda, Cestoda, Myrmicinosporidium, Mattesia, EvoDevo

## Abstract

As social insects, ants represent extremely interaction-rich biological systems shaped by tightly integrated social structures and constant mutual exchange with a multitude of internal and external environmental factors. Due to this high level of ecological interconnection, ant colonies can harbour a diverse array of parasites and pathogens, many of which are known to interfere with the delicate processes of ontogeny and caste differentiation and induce phenotypic changes in their hosts. Despite their often striking nature, parasite-induced changes to host development and morphology have hitherto been largely overlooked in the context of ecological evolutionary developmental biology (EcoEvoDevo). Parasitogenic morphologies in ants can, however, serve as “natural experiments” that may shed light on mechanisms and pathways relevant to host development, plasticity or robustness under environmental perturbations, colony-level effects and caste evolution. By assessing case studies of parasites causing morphological changes in their ant hosts, from the eighteenth century to current research, this review article presents a first overview of relevant host and parasite taxa. Hypotheses about the underlying developmental and evolutionary mechanisms, and open questions for further research are discussed. This will contribute towards highlighting the importance of parasites of social insects for both biological theory and empirical research and facilitate future interdisciplinary work at the interface of myrmecology, parasitology, and the EcoEvoDevo framework.

## Introduction

Within the EcoEvoDevo framework, organisms are considered parts of complex webs of ecological interactions. Within these systems, the environment plays a crucial and influential role that may shape ontogenetic and evolutionary trajectories, not only via such factors as temperature, chemicals or interactions with conspecifics, but also by way of symbionts, microbiomes, pathogens, and parasites [1–6]. An organism’s environment may thus be the source and inducer of genotypic and phenotypic variation, while development acts as a regulator that can mask, release, or create new combinations of variation, and novel phenotypes may arise when these variations are subsequently fixed by natural selection [1]. In short, within this theoretical context “The environment is not merely a permissive factor in development. It can also be instructive” ([7]:8).

As social insects, ants represent one of the most interaction-rich biological systems, their existence shaped by tightly integrated, superorganismal social structures interacting with predators, prey, mutualistic microbes and pathogens alike [5]. Due to this extremely high level of interconnectedness with their external and social environment on the one hand, and their complex holometabolous development leading to highly specialized caste-specific phenotypes on the other [8–10], ants are featured as key study organisms in a number of publications investigating the interactions of evolution, ecology and development: ant model systems have been used to study environmental effects on developmental modularity and robustness [11, 12], caste determination [10, 13], caste ratio [14–16], and the induction of seemingly lost ancestral developmental pathways [9, 10]. Even major transitions in the realms of eusociality and individual ontogeny may have been mediated by EcoEvoDevo interactions on multiple levels, involving environmental, genetic and epigenetic influences on embryonic development, caste evolution and colony organization [5, 6, 17].

Furthermore, social insect colonies are known to harbour an extremely diverse community of parasites and parasitoids [18–22], which constitute a part of their external environment [5]. For ants alone, current scientific publications yield over 1400 records of parasites and parasitoids from 51 families infecting 82 genera of ants [22, 23]. This vast number of host–parasite systems has yielded a large amount of publications addressing diverse topics, from the impact of the host’s ecology and life history on its susceptibility to parasitism (reviewed in [22]) to social immunity [24–27], pest management [28] and untangling the phylogenies of ant hosts and their behaviour-altering “zombie” parasites [29]. It therefore becomes apparent that interactions with parasites constitute a significant part of social insects’ ecological interconnections that may have far-reaching effects on both individual and colony. This is especially evident in the rather small portion of parasites infecting ants at the larval or pupal stage, which have the ability to influence their hosts’ adult morphology—from cuticle colour to caste identity. The prevalence of especially extensive parasite-induced morphologies in ants has been attributed to both their high degree of phenotypic plasticity as well as mechanisms of colonial buffering, allowing even strongly modified specimens to survive within the colony [11, 12] (see also “Discussion”). The resulting parasitogenic phenotypes can be viewed as “natural experiments” brought about by environmentally induced changes in development that may provide insight into the underlying mechanisms of ontogeny, physiology and caste differentiation [11, 30–33].

In stark contrast to the well-studied relevance of symbiotic organisms (e.g., [3, 6])—the influence of parasites on host development has remained largely overlooked within the existing EcoEvoDevo literature until now. Despite having long been recognized as potential “developmental switches” with multidimensional effects [34, 35] on host phenotypes [11, 30, 31, 36, 37], parasitic organisms often appear as little more than a side-note in previous publications (e.g., [1, 2, 4, 5]) and the discussion of their role in the development and evolution of their hosts is mostly restricted to brief mentions of the “extended phenotype” concept [38, 39]. Surprisingly, however, older works of scientific literature—mainly written in German and therefore inaccessible to many researchers—draw remarkably enlightened conclusions about the connections between environmental factors (such as nutrition and the timing of parasitic infection), larval development, and effects on the adult phenotype [30, 37, 40]. Incorporating these accounts herein, as well as translating and discussing them in light of current biological theory (Laciny, Abouheif, Wheeler, Metzl, in prep.) will provide additional insights into the history of the EcoEvoDevo school of thought and the relevance of parasitogenic phenotypes within it.

The vast body of literature treating ants and their parasites presents yet another unexpected knowledge gap: certain research foci being more prevalent than others, the topics of pest management (e.g., [28]), social immunity (reviewed in [26]), and behavioural alteration by “zombie” parasites (e.g., [23, 29, 41, 42]) are especially well-researched and currently of interest to many scientists. Several recently published review papers have treated the parasite community of selected ant genera [21], biodiversity of ant parasites [22], and the behavioural consequences of parasitism [23]. Morphological aberrations due to parasites have albeit been somewhat neglected: ants as interaction-rich, polymorphic, holometabolous insects often infected during the larval or pupal stage, ants provide numerous case studies about parasitic influence on development, phenotype and caste identity. However, comparative descriptions of these morphological phenomena have not been the subject of a focussed literature review study to date. Myrmecologists working in the field or in natural history collections are thus in need of an organized overview of the most common morphological aberrations and the parasites that cause them, to aid in the recognition and further study of these rare specimens. Likewise, researchers of evolutionary, developmental and theoretical biology will find the world of ants and their parasites to provide ample inspiration and opportunity for the study of hitherto unknown ontogenetic mechanisms, triggers and pathways beyond currently established model organisms.

This article thereby aims to bridge the currently existing gap between the worlds of the EcoEvoDevo framework and morphology-based parasitology of ant hosts. By presenting a review of parasitogenic phenotypes in the most relevant host–parasite systems, as well as identifying knowledge gaps and opportunities for further studies, I wish to complement the subjects of myrmecology, parasitology, and EvoDevo alike. In advocating to combine these hitherto separate realms in an interdisciplinary manner, I ultimately hope to better integrate ant–parasite systems into the EcoEvoDevo framework as powerful agents of developmental and evolutionary change.

## Methods

### Selection of host–parasite systems

Among the plethora of parasites and pathogens known from ant colonies [22, 23], only a limited subset is able and known to influence host development and thereby cause morphological aberrations in adult ants. Selected taxa treated within this study must thus meet the following criteria:

- Extant host and parasite taxa.
- Preimaginal infection (egg, larval, or pupal stage) of ant host.
- Completion of imaginal development, survival of host until eclosion.
- Identifiable morphological changes to external or internal structures of the host beyond mere visible presence of parasite (e.g., physogastry, visible spores).

This method of selection thus excludes many well-studied and charismatic parasites which obviously cause observable changes in appearance and behaviour but only infect ants after they have already reached the imaginal stage, e.g., *Ophiocordyceps* “zombie” fungi [29, 41], most “ant decapitating” Diptera [43], or the river fluke *Dicrocoelium dendriticum* [44]. It further excludes all parasites which infect juvenile stages, but cause no known changes to morphological structures (e.g., males of myrmecolacid Strepsiptera, [45]), host–parasite relationships only known from fossils (e.g., ants and *Heydenius* spp. nematodes [42]), and parasitoids which infect juvenile stages but cause host death before imaginal development is completed (e.g., many hymenopteran parasitoids, [46]). Certain ant-associated bacteria, such as *Blochmannia* and *Wolbachia* undoubtedly play important roles in ant development and evolution [6, 47] and may affect morphology and colony composition [48, 49]. However, they are generally characterized as endosymbionts rather than parasites and would therefore exceed the scope of this paper.

Based on these criteria, all described morphological alterations to the host phenotype in the included host–parasite systems can be interpreted as changes caused by parasitic disruption of typical developmental patterns during host ontogeny and/or metamorphosis. Known parasite-induced changes to host behaviour (reviewed in [23]) are included for completeness, though the focus of the present paper is placed on morphological changes. In some questionable cases, where currently available literature data do not yet allow clear conclusions to be drawn, taxa are included, but their compliance with the criteria above is discussed.

Selected parasite taxa meeting these criteria in at least one previously documented case and described in the literature in sufficient detail to be treated within this study are therefore:

1. Nematoda:
  1.1 Mermithidae
  1.2 Tetradonematidae
  1.3 Allantonematidae, Physalopteridae, Seuratidae
2. Cestoda
  2.1 Davaineidae
  2.2 Dilepididae
3. Apicomplexa: Neogregarinorida: *Mattesia* spp.
4. Fungi: *Myrmicinosporidium durum*
5. Viruses (?): “labial gland disease”

### Data collection

Targeted manual reference search was conducted via Google Scholar, the Biodiversity Heritage Library (https://www.biodiversitylibrary.org), and the Zoological Library at the Natural History Museum, Vienna. This mode of data collection was chosen to facilitate the inclusion of a diverse spectrum of historical and linguistically diverse publications, as well as those published in smaller, unlisted journals, which may remain undetected by automated search protocols. Literature included herein was subsequently assembled based on the references of recent thematically relevant review papers [21–23], comparative historical accounts (e.g., [31, 50]), as well as further references cited within the respective case studies, and the author’s previous work [51–53]. In total, ca. 120 publications containing descriptions of parasitically altered host morphology, dating from 1747 to 2021, were used to collect relevant data for this study. Specifically, papers were ordered by parasite group and subsequently assessed for the following information:

Author(s) and date of publication, current taxonomic placement of the parasite, current taxonomic placement of the host, locality, host caste (but see caveats below), life stage of host at infection, description of host morphology, (putative) mechanisms underlying parasite-induced changes, and further biological information (e.g., host behaviour, additional ecological factors).

The most commonly encountered parasite-induced phenotypic traits are summarized as “syndromes” in Table 1; for consistency and easy overview, the corresponding abbreviations can be found in the overall summary provided in Table 2. More detailed accounts are given in the descriptions of the respective host–parasite systems, and the main traits and processes implicated in the literature as relevant interactions between parasitism and host development are summarized in Fig. 8.

**Table 1:** Abbreviations and descriptions of five main morphological syndromes commonly encountered in parasite-induced ant phenotypes

Code	Syndrome	Description
CU	Cuticle	Altered colour, thickness, pilosity or sculpture of the cuticle compared to healthy conspecifics
IC	Intercaste	Morphology combining characteristics of two or more healthy castes of same species
PG	Physogastry	Gaster enlarged or distended due to presence of a parasite
PR	Proportions	Altered proportions of morphological structures (e.g., appendage length, head width) compared to healthy conspecifics of same caste
SXR	Reduced sex characters	Reduction of gonads, ocelli, wings or thoracic sclerites in specimens otherwise corresponding to gyne or male morphology

**Table 2:** Summary of all featured parasite groups in order of appearance in the text, including host taxa (in alphabetical order, multiple affected species per genus denoted as spp.), host castes (m—male, w—worker, s—soldier, q—queen/gyne, IC—intercaste), regions of occurrence and elicited morphological changes as defined in Table 1; doubtful cases marked with *

	Parasite taxa	Host taxa	Host castes	Regions	Syndromes
Nematoda	Mermithidae: *Pheromermis*, *Agamomermis*, *Allomermis*, *Camponotimermis*, *Hexamermis*, “*Mermis*”*, *Meximermis*	*Aphaenogaster subterranea*, *Camponotus* spp., *Cephalotes minutus*, *Colobopsis* spp., *Eciton burchellii*, *Ectatomma* spp., *Lasius* spp., *Myrmica* spp., *Odontomachus* spp., *Pachycondyla* spp., *Pheidole* spp., *Polyrhachis* spp., *Solenopsis* spp., *Tetramorium caespitum*	m, w*, s*, q, IC	Europe, USA, South America, Australia, Papua New Guinea, D. R. Congo, Borneo	CU, PG, IC, PR, SXR
	Tetradonematidae: *Myrmeconema neotropicum*	*Cephalotes atratus*	w	Panama, Peru	CU, PR
	Tetradonematidae: Tetradonema solenopsis*	*Solenopsis invicta*	w	Brazil	CU
	Allantonematidae: *Formicitylenchus oregonensis*	*Camponotus vicinus*	q	USA	SXR
	Seuratidae: Rabbium paradoxus*	*Camponotus castaneus*	w	USA	PG
	Physalopteridae: *Skrjabinoptera phrynosoma*	*Pogonomyrmex barbatus*	w*	USA	CU, PG
Cestoda	Davainaeidae: *Raillietina* spp., *Cotugnia* spp.	*Leptothorax* spp., *Monomorium* spp., *Myrmica* spp., *Pachycondyla sennaarensis, Pheidole* spp., *Tetramorium* spp., Formica rufa*	m, w, s, q	Europe, USA, India, Australia, Sudan	CU
	Dilepididae: *Anomotaenia* spp., *Choanotaenia* spp.	*Harpagoxenus sublaevis*, *Leptothorax acervorum*, *Temnothorax* spp.	m, w, s, q, IC	Europe, USA	CU, IC, PR, SXR
Apicomplexa	Neogregarinorida: Lipotrophidae: *Mattesia* sp.	*Harpagoxenus sublaevis, Leptothorax* spp., *Monomorium pharaonis, Myrmecia* spp.*, Myrmica rubra*, *Solenopsis* spp., *Temnothorax* spp.	m, w, q	Canada, USA, Europe, Brazil, Australia*	CU
Fungi (inc. sed.)	*Myrmicinosporidium durum*	*Aphaenogaster senilis*, *Cardiocondyla elegans*, *Cataglyphis hispanica*, *Crematogaster auberti*, *Goniomma hispanicum*, *Strongylognathus ceciliae*, *Messor barbarus*, *Nylanderia vividula*, *Pheidole* spp., *Plagiolepis* spp., *Pogonomyrmex* spp., *Solenopsis* spp., *Strumigenys membranifera*, *Tapinoma* spp., *Temnothorax* spp., *Tetramorium* spp.	m, w, s, q	USA, Europe, Japan, Galapagos Is	CU, PG
Virus (?)*	“Labial Gland Disease”	*Camponotus* spp., *Formica* spp., *Prenolepis* spp.	m, w, q	USA, Europe, Japan	CU, IC, SXR

Due to the large proportion of historical literature on the one hand and the scarcity of data on some of the included host–parasite systems on the other, standardization and comparability across studies proved challenging. To the best of her knowledge, the author has provided the correct taxonomic information on hosts and parasites at least to family level, as they correspond to the current state of research. For most host–parasite systems featured herein, empirical data on the causal mechanisms linking parasitism to the observable morphological changes are still severely lacking or currently matters of scientific dispute (e.g., [32, 33]). The author has thus opted to present contesting hypotheses about causal factors wherever appropriate and highlight these knowledge gaps to inspire further research. Any obviously outdated or erroneous information from previously published works is discussed where relevant and rectified wherever possible.

### Notes on caste- and size-related terminology

While reviewing the body of scientific literature for the present publication, the author became aware of certain methodological and terminological discrepancies that may hamper comparability of studies and usability of data for future research if left uncommented. Within the literature surrounding parasite-induced morphologies, these issues particularly concern the language employed when describing shifts in host size and caste identity.

The morphological diversity of known parasitogenic phenotypes—especially in ants with polymorphic caste systems—has led to the assumption that parasitogenic phenotypes develop directly from the caste they are most similar to [50]—a claim contested in more recent publications (e.g., [33]). Within the present publication, the author has opted to follow the respective literature cited when referring to host castes. This necessitates the caveat that these terms only have merit for comparative purposes and refer to superficial resemblance rather than ontogenetic origin. Cases where multiple sources are contradictory or newer evidence refutes historical caste assumptions are discussed wherever appropriate.

Closely related to the subject of caste identity is the interpretation of morphological proportions under parasitic influence: throughout the present work, morphological structures or entire bodies of the host will be referred to by terms such as “hypertrophied”, “reduced”, “elongated” or “shortened”. It is crucial to clarify that these terms are always relative and comparative in nature. Though not always the case in the literature cited, the author has made an effort to specify the frame of reference (i.e. the assumed “original” caste used for comparison) for any affected structure. Controversial cases found in the literature will be highlighted within the respective chapters and further ramifications of this issue are commented in “Discussion”.

## Parasite groups

### Nematoda

#### Mermithidae

**Fig. 1:**
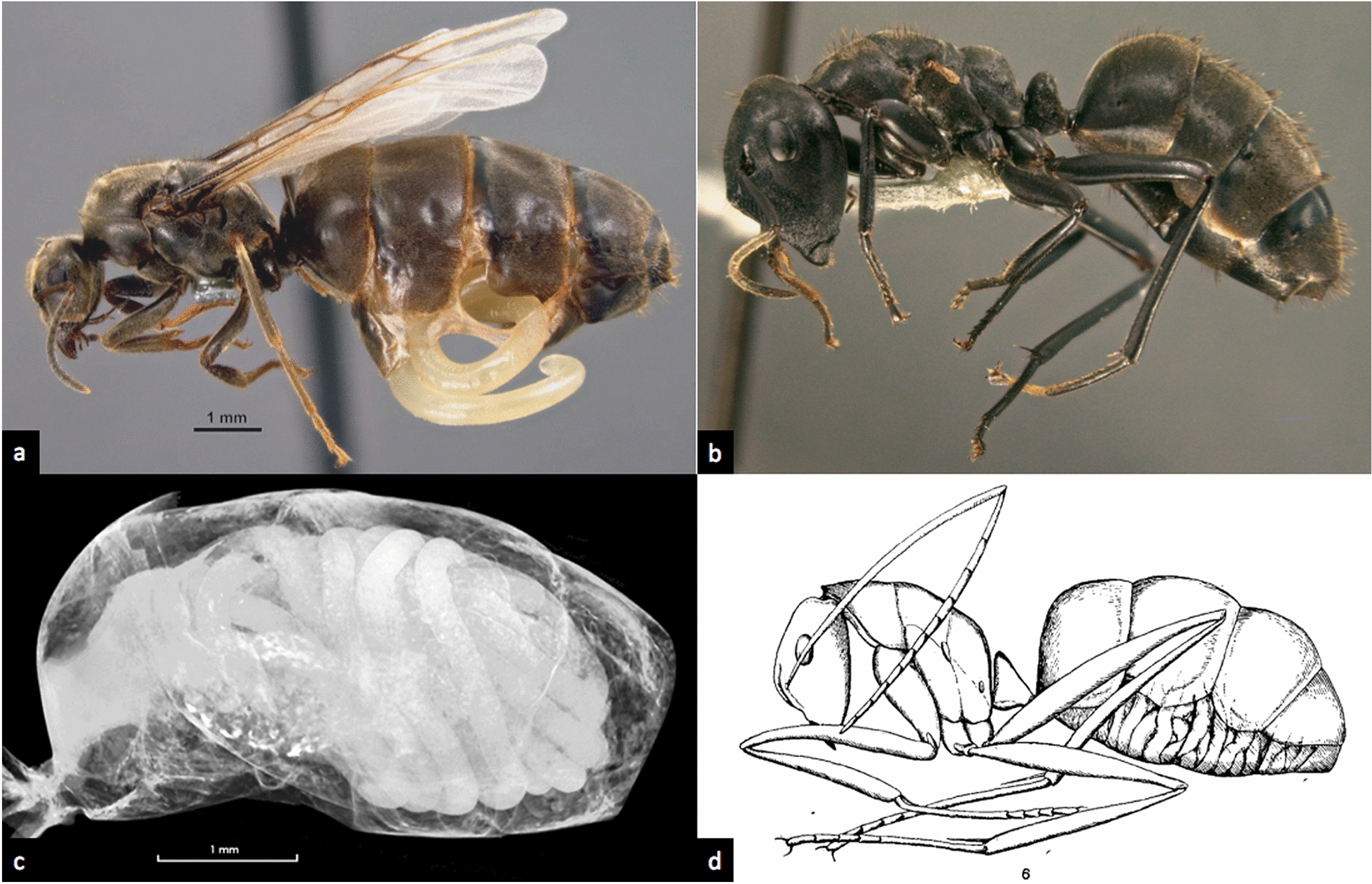
Ants as hosts of Mermithidae: **a** *Lasius niger* mermithogyne with mermithid erupting from gaster, note shortened wings (from [51]); **b** *Colobopsis* sp. “nrSA”, mermithogenic intercaste, note wing stubs and black colour (from [52]); **c** microCT image of same specimen, mermithid visible in gaster (from [52]); **d** *Camponotus pompeijus* mermithergate, mermithid visible in gaster (from [50])

Among the parasites known to elicit morphological changes in their ant hosts, surely the longest research tradition and most extensive body of work surrounds the nematode family Mermithidae (Fig. 1). Mermithid nematodes occur world-wide and are common macroscopic endoparasites of arthropods, including most subfamilies of the Formicidae. To date, six extant genera of the family Mermithidae are known to parasitize ants [42]. The first published scientific record of ants parasitized by mermithid nematodes dates back to Gould [54], who described long, white worms from “large and small ant-flies” (i.e. alate gynes and males) as early as 1747. Later, especially researchers of the early twentieth century (Fig. 1d) showed a growing scientific interest in these parasites and the often bizarre morphologies produced in their ant hosts (e.g., [30, 36, 37, 50, 55–67]).

Many studies have investigated parasitogenic effects in the Formicinae and Myrmicinae, especially the genera *Lasius* (e.g., [30, 37, 51, 55, 64, 65, 68, 69]) and *Myrmica* (e.g., [33, 70–74]). While these taxa may be among the most ubiquitous and commonly infected, other accounts report mermithid infections of Ponerinae, Ectatomminae and Dorylinae [50, 57, 60, 66, 75] as well as of charismatic groups like the Southeast-Asian “exploding ants” (*Colob-opsis* spp., [52, 53]) or invasive species like *Solenopsis invicta* [76, 77].

In the few well-studied cases, mermithids develop in an indirect life-cycle involving paratenic (intermediate) hosts in moist environments (e.g., oligochaetes or aquatic insect larvae in *Pheromermis* spp.), which contain the infective nematode juveniles and are fed to ant larvae as a protein source [68, 78]. Subsequently, the nematode and the infected ant larva develop in synchronicity until eclosion of the ant imago. One ant host usually contains a single mermithid, but up to nine nematodes per host have been reported [77]. When the mermithid has reached maturity, it will eventually alter the infected ant’s behaviour, leading to host suicide by drowning, to release the parasite [42, 55, 68, 79].

Parasitized individuals can present with a wide range of aberrant characters and proportions: while male hosts may exhibit slight shifts in size, allometry and gonad development [30, 33, 37, 69, 76], mermithid nematodes are known to cause intercaste or “mosaic” (sensu [11]) phenotypes in female ants: these may present anywhere on a wide spectrum of possible morphologies and can resemble workers, soldiers, gynes, possess combinations of the healthy castes’ characters or exhibit entirely novel traits [33, 50, 52, 68, 80, 81]. In comparison to the respective original host caste, characteristic changes may include altered body size, elongated or shortened extremities, physogastry (enlarged gaster, distended by the parasite), reduced size of head, deviations in pilosity and sculpture, as well as reduction of all sexual characters (wings, thoracic sclerites, ovaries, and ocelli; Fig. 1a, b) (e.g., [31, 33, 42, 50–52, 55, 67–71, 74]).

The extent of morphological alterations induced by mermithid infections can thus range from no observable changes apart from slight physogastry (e.g., in *Solenopsis* spp., [76, 77, 82]) to aberrations extreme enough to render morphology-based caste or even species assignment impossible (e.g., in *Myrmica* spp. or *Colobopsis* sp., [33, 52]. Unsurprisingly, mermithogenic phenotypes have led to instances of taxonomic confusion in the past, because parasitized individuals were mistakenly described as new taxa on several occasions [70–73].

This diversity of phenotypes has led to the use of specialized terminology, such as “intermorph/intercaste”, “mermithogyne” (infected gyne or queen), “mermithergate” (infected worker), “mermithostratiote” (infected soldier), or “mermithaner” (infected male) to describe these specimens [32, 50, 81]. Originally, these categories were based on the assumption that mermithogenic phenotypes develop directly from the caste they are morphologically most similar to [50]. In contrast, newer studies on *Myrmica* spp. have proposed a common origin of all aberrant morphologies from larvae destined to become gynes or males [33] or opted to omit caste assignment of the host in light of unclear morphology [74]. Accounts of “workers” and “soldiers” exhibiting gigantism or gyne-like traits [32, 36, 83] or infection of adult ants [63, 67] are currently considered doubtful and are in need of further investigation.

Mermithids themselves are only reliably identifiable morphologically in their rarely encountered mature stage [84, 85]. Attempts to recreate their life-cycles under controlled laboratory conditions in order to rear mature specimens have been largely unsuccessful [69]. Many hitherto published studies have therefore had to forgo identifying parasites to species or even genus level and settle for a family-level identification (Mermithidae) (e.g., [52, 76, 83]) or the largely outdated genus name “*Mermis*” instead (e.g., [50, 73, 79]). Due to this often unresolved parasite taxonomy but comparable variability of morphological syndromes across identified taxa, Mermithidae are summarized at the family level in Table 2.

The mechanisms whereby mermithid nematodes influence host phenotypes have long been a matter of speculation; historical hypotheses range from larval hypertrophy by overfeeding ([36, 83], now considered outdated, see [30, 33]) to hormonal or chemical influences [60, 69]. The currently most common hypothesis assumes nutrient depletion through metabolic competition between host’s and parasite’s tissues during preimaginal development [30, 33, 37, 60, 70, 71, 86]. This model considers the importance of timing and severity of infection and interprets morphological changes as results of metabolic disturbances during ontogeny. For gynes of *Myrmica* and *Lasius*, Kloft [37] describes a consistent sequence, in which mermithids deplete pupal energy reserves of their hosts via hydrolysis of tissues: first, the flight musculature is replaced by loose fatty tissue, followed by depletion of the gastral fat body and, finally, the gonads.

Whether the extent of the changes to host morphology mainly depends on timing of infection, size, and number of the parasites, or whether combinations of different host and parasite taxa result in different levels of developmental robustness or plasticity [11, 33, 53] must be further investigated. Thus, despite the plethora of literature available on the ant–mermithid system, it still offers numerous open questions and opportunities for further research (see “Discussion”, Outlook).

#### Tetradonematidae

From the family of tetradonematid nematodes, only two species are known to cause morphological aberrations in ants:

*Tetradonema solenopsis*, the first tetradonematid parasite to be discovered in ant hosts, was described from the host ant *Solenopsis invicta* in Brazil [87]. Infected workers were reported to have enlarged gasters and scalloped gastral tergites. Due to the role of *S. invicta* as an agricultural pest, *T. solenopsis* has been discussed as a possible biological control agent [28]. However, as the parasite’s life-cycle and the timing of infection are unstudied in this case, it is unknown whether the observed changes in morphology represent the results of developmental disturbances or are simply due to the presence of the parasite in the adult host [22, 42].

**Fig. 2:**
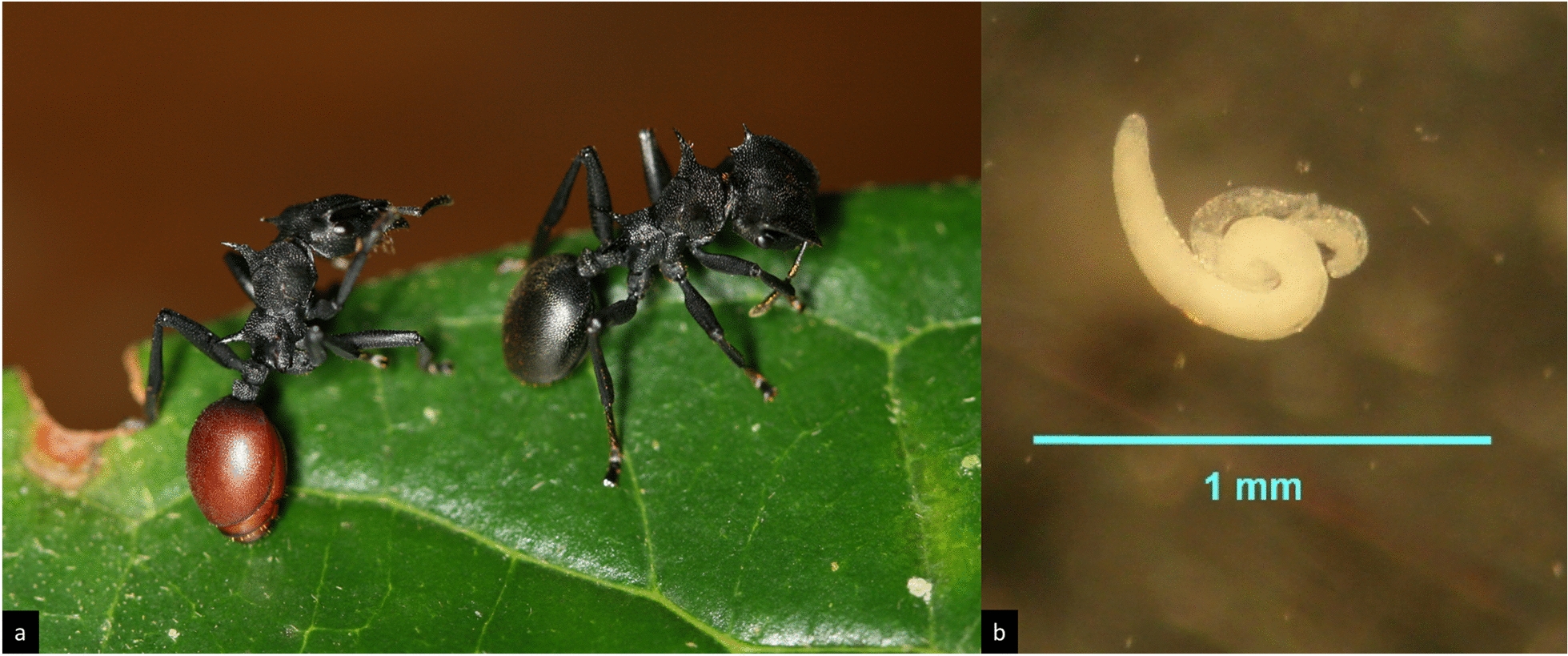
*Cephalotes atratus* infected by *Myrmeconema neotropicum*: **a** infected *C. atratus* worker with berry-like gaster (left), healthy worker (right); **b** *M. neotropicum* mating pair (male top, female bottom) (photos: S.P. Yanoviak)

*Myrmeconema neotropicum* is perhaps one of the most charismatic parasites known from ants: in its only known hosts, workers of the neotropical arboreal ant *Cephalotes atratus*, it causes a conspicuous change in the colour of the gaster from black to shiny red (Fig. 2a) [88, 89]. This parasite-induced colour morph has been known for more than 100 years, but was erroneously described as the separate taxon *C. atratus* var. *rufiventris* [90]. The nematode infects the ant host at the larval stage via eggs or larvae of the parasite contained in bird faeces [91]. Developmental stages of *M. neotropicum* can thus be found in all life stages of the ant, with mating adult parasites (Fig. 2b) present in callow workers and fertilized females in adult ants exhibiting a red gaster [88]. The reddish colour of the gaster can extend to the femoral integument in late stages of infection and is thought to be caused by a parasite-induced thinning of the cuticle, which reaches its most noticeable appearance when the eggs mature and the parasite is most infective [91]. In addition to this eye-catching colour change, infected ants also exhibit atrophy of the ventral nerve cord [89], a weakened attachment of the gaster at the postpetiole, reduction of head size by an average of 10%, an increase of gastral mass and a decrease in overall body mass (excluding gaster) [88, 92]. Interestingly, despite the increase in gastral weight, studies found a decrease in metabolic rate of the gastral tissue in parasitized ants [93]. Apart from these morphological changes, infected *C. atratus* ants show altered behavioural patterns, acting more sluggish and less aggressive than their healthy nestmates—which has been attributed to lower levels of alarm pheromones [23, 88]—as well as a peculiar gaster-flagging display. These behavioural traits combined with the red, berry-like, and weakly attached gaster have led to the hypothesis of “fruit mimicry” [89], whereby the parasite-induced changes to the phenotype serve to attract birds, which devour the infective gasters and thus complete the parasite’s life-cycle. The *M. neotropicum–C. atratum* system has thus become one of the textbook examples of the so-called extended phenotype concept, wherein changes to the host phenotype may serve to increase parasite fitness [38, 92] (see also “Discussion”).

#### Other Nematoda

**Fig. 3:**
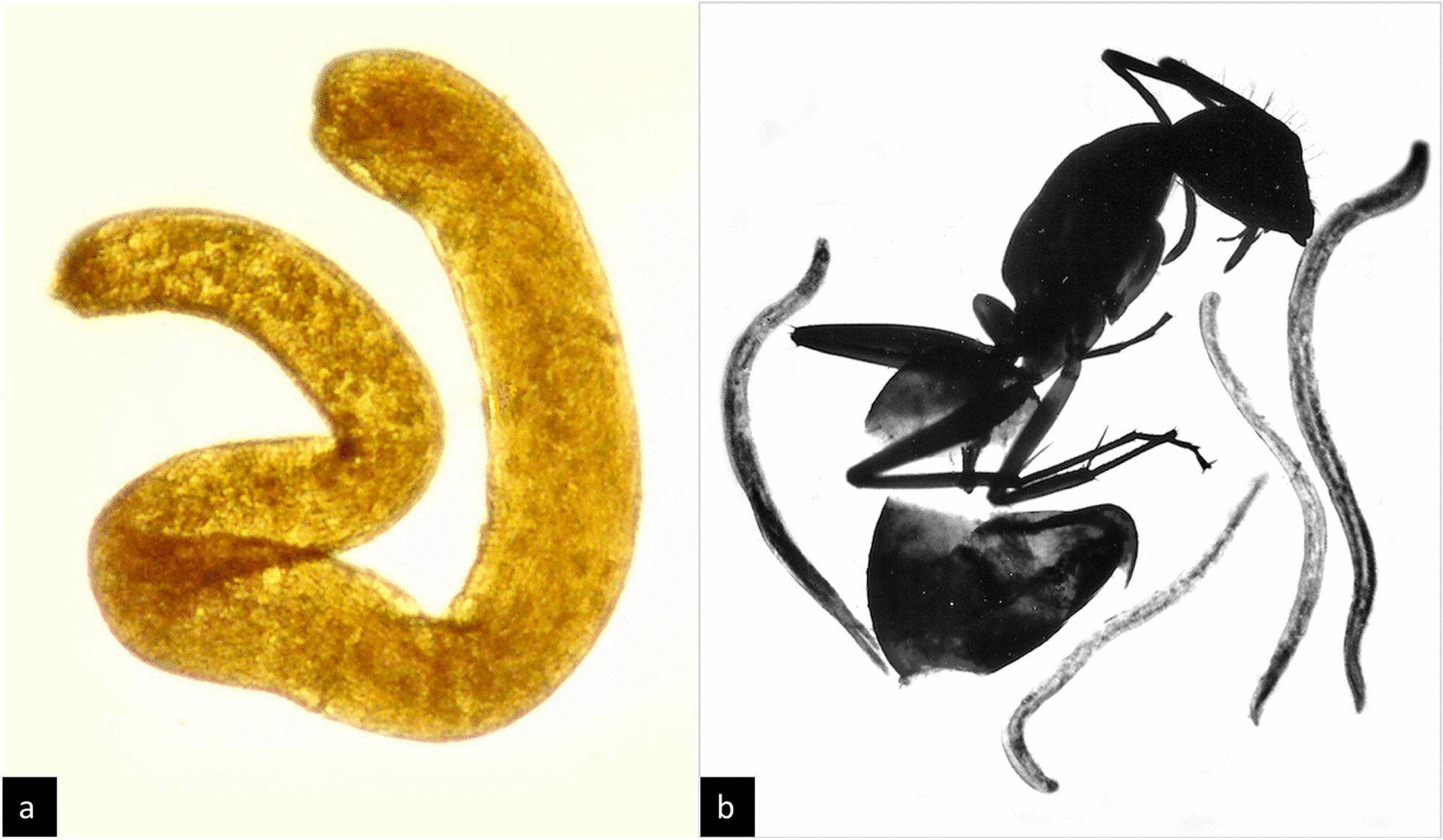
Ant–parasitic Nematoda of the families Allantonematidae (**a**) and Seuratidae (**b**): **a** *Formicitylenchus oregonensis* from host *Camponotus vicinus*; **b** *Rabbium paradoxus* adults with host *Camponotus castaneus* (photos from [42], provided by G. Poinar)

Apart from the occurrences of the relatively well-studied mermithid and tetradonematid nematodes described above, members of three other families of the Nematoda are mentioned sporadically as parasites with possible phenotypic effects on their ant hosts (reviewed in [42]):

Within the Allantonematidae, *Formicitylenchus oregonensis* is reported as a parasite of queens of *Camponotus vicinus* from Oregon, USA (Fig. 3a). Poinar [94] reports one adult female and 120 juveniles of the parasite found in the body cavity of the dealate gyne host. The infected ant exhibited reduced, abnormally formed ovaries and eggs. While the parasite’s life-cycle remains unknown, the author hypothesizes infection through the host larva’s cuticle and a possible dispersal of the parasite during the nuptial flight of winged *Camponotus* queens.

A case of ant parasitism by a nematode of the family Physalopteridae is illustrated by Lee [95], who first reported *Skrjabinoptera phrynosoma* from *Pogonomyrmex barbatus* occurring in Texas, USA. Infected worker ants are recognizable by their enlarged, light-coloured gaster. In the complex cycle, the ants represent the intermediate host for this nematode parasite of the Texas horned toad (*Phrynosoma cornutum*): dead, gravid female nematodes expelled by the final host are an attractive food source for the ants and are fed to ant larvae. During the ants’ larval and pupal stage, the juvenile nematodes develop and eventually encyst in the host’s fat body (up to 75 cysts per host). When infected ants are eaten by the final host lizards, parasite development is completed.

A single questionable case of parasite-induced host phenotype is reported from the Seuratidae, with *Rabbium paradoxus* infecting *Camponotus castaneus* workers in Florida, USA (Fig. 3b) [96]. While no infection of juvenile ants is known, infected workers exhibit an enlarged gaster and behavioural shifts to more diurnal activity, possibly facilitating vertebrate predation. Interestingly, this host–parasite pair may be currently in transition between an indirect cycle involving a final vertebrate host and reproduction of the parasite entirely within the infected ant [42].

### Cestoda

#### Davaineidae

Tapeworms of the davaineid genera *Cotugnia* and *Raillietina* are known to utilize ants and other arthropods as intermediate hosts before infecting their final hosts, several species of birds and mammals, e.g., grouse, chickens, turkeys, emus, and rabbits [97–106]. Workers, soldiers, gynes, and males of the myrmicine genera *Pheidole*, *Tetramorium*, *Monomorium*, *Leptothorax*, *Pachycondyla*, and *Myrmica* [97–100, 102, 104, 107, 108] have been identified as intermediate hosts containing cystercercoids. *Formica rufa*, reported to harbour *Raillietina *friedbergeri and thereby the sole published formicine host of davaineid cestodes, is listed as “not experimentally verified” [107].

The role of these cestodes as parasites of economically important animals has contributed to the rather extensive body of literature surrounding them. However, detailed investigations of morphological aberrations in ant hosts are extremely sparse: apart from cystercercoids (up to 50 per host, see [99]) visible though the gastral integument [100], only a darker colour of the cuticle has been reported as a suspected parasite-induced alteration of the host phenotype. An account of this phenomenon along with a hypothesis for its origin in *Myrmica rubra* and *M. scabrinodis* infected with *Raillietina urogalli* is provided by Muir ([99]: 689): “The cysticercoids have been found in males, queens and workers of both species, the infected ants being detected by an unnaturally dark chocolate colouration affecting the whole cuticle, compared with the dark reddish-brown tint of non-infected individuals. This colour difference may be due to the formation of a melanoid pigment from the excretions of the parasite.”

#### Dilepididae

Among cestodes as parasites of ants, the greatest number of publications treats the family Dilepididae (Cestoda, Cyclophyllidea). The species *Choanotaenia unicoronata* [109] and—more commonly—*Anomotaenia brevis* [110–114] have been identified as parasites of ants, while in several cases in the literature, the parasites remain determined only to the generic or family level (e.g., [40, 115, 116]). Cestode eggs are taken up by ant larvae, presumably from the faeces of several bird species (e.g., woodpeckers, quail), which represent the final hosts (see Fig. 1 in [114]).

Infected ants reported from throughout Europe, northern Africa and the USA [115] belong exclusively to the subfamily Myrmicinae, comprising several species of Temnothorax, as well as *Leptothorax acervorum* and its slavemaker *Harpagoxenus sublaevis* (see Table 2).

The majority (up to 90% [111]) of infected ants were identified as workers (but see “Discussion”), with several authors also reporting lower rates of infection in gynes and males, and the occurrence of presumably parasite-induced intercaste phenotypes [40, 111, 112]. The number of cystercercoids found in the gaster of each parasitized individual varied greatly from one to over 100 [40].

**Fig. 4:**
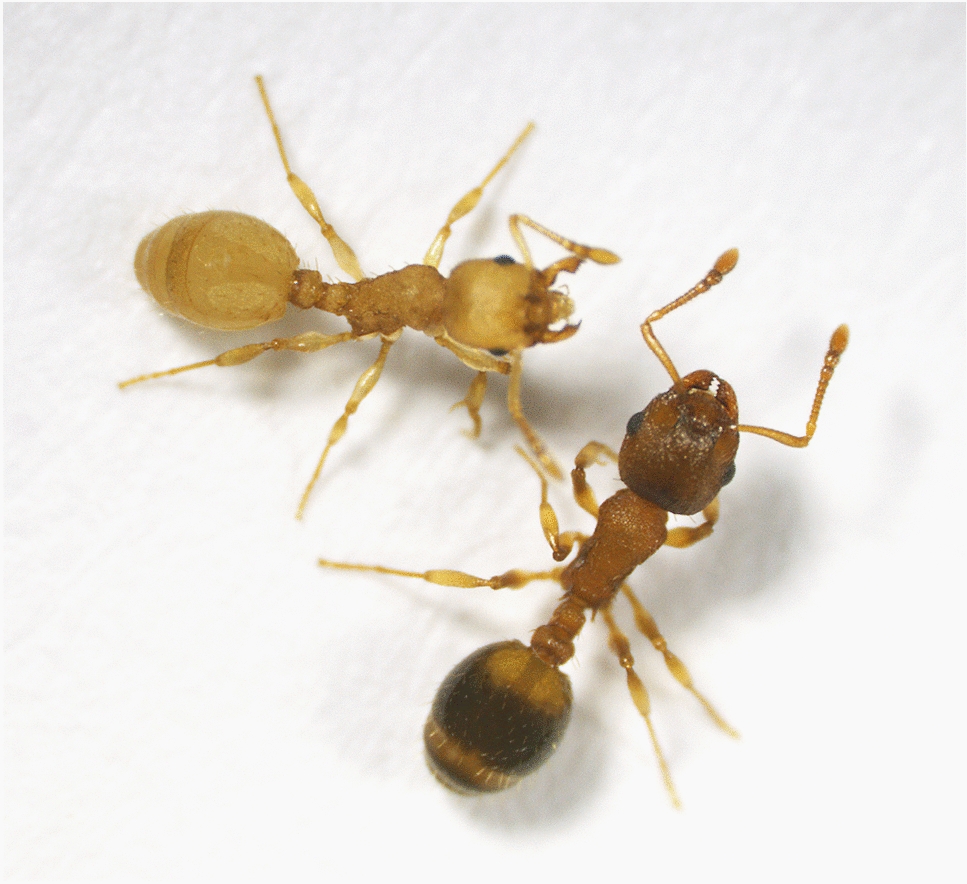
*Temnothorax nylanderi* infected by the dilepidid cestode *Anomotaenia brevis*; infected worker (top, note yellow colour) with healthy nestmate (bottom) (photo: S. Foitzik)

Infection coincides with certain characteristic morphological changes in the host (Fig. 4): a yellowish and unusually soft cuticle, widening of the petiole and postpetiole, shortened antenna, tibia and femur, reduction of head size and overall body size, and atrophy of mandibular muscles in workers, as well as lowered fertility and intercaste morphology in presumptive gynes [40, 109–120]. While the exact developmental mechanisms underlying these changes are unknown, authors have hypothesized disruptions of imaginal disks and hormone levels, depletion of melanin precursors, and malnutrition during the larval or pupal phase as possible causes [40, 110, 111, 118].

In addition to morphological alterations, some studies report increased longevity [114] and changes in behaviour, especially sluggish movement, increased begging for food and reduced aggression in workers, and less flight activity in gynes [40, 110, 112, 116, 118]. These alterations to social and overall behaviour of infected ants are thought to be connected to observed changes in the ants’ cuticular hydrocarbon (CHC) profile [111, 113, 114]. Interestingly, infection of some individuals within a colony seems to have an effect on uninfected nestmates as well: even though infected colonies do not seem to suffer significant production or fitness losses, they may produce fewer eggs while investing in more or bigger males, while uninfected workers display reduced aggression and increased mortality rates during periods of colony stress [112, 113, 121]. Upon removal of the queen, infected *Temnothorax nylanderi* workers showed increased reproductive potential compared to their healthy nurse sisters [122]. These complex interactions of parasitism, behaviour, reproduction and colony composition have been interpreted as mechanisms of colonial buffering [11, 112, 113, 122].

Recent studies comparing gene expression in *T. nylanderi* parasitized by *A. brevis* to healthy conspecifics [114, 123] found differences in expression patterns of over 400 genes, many linked to cuticular hardening, CHCs, metabolism, lifespan, fertility, and muscle function, and found no evidence of neurochemical influences on host behaviour by the parasite. The authors interpret this parasitogenic syndrome—particularly cuticular softening, altered colouration and reduced activity—as traits that may facilitate parasite transmission to the final woodpecker host, interpreted as an example of the extended phenotype concept (sensu [38], but see “Discussion”).

### Apicomplexa: Neogregarinorida: *Mattesia* spp.

**Fig. 5:**
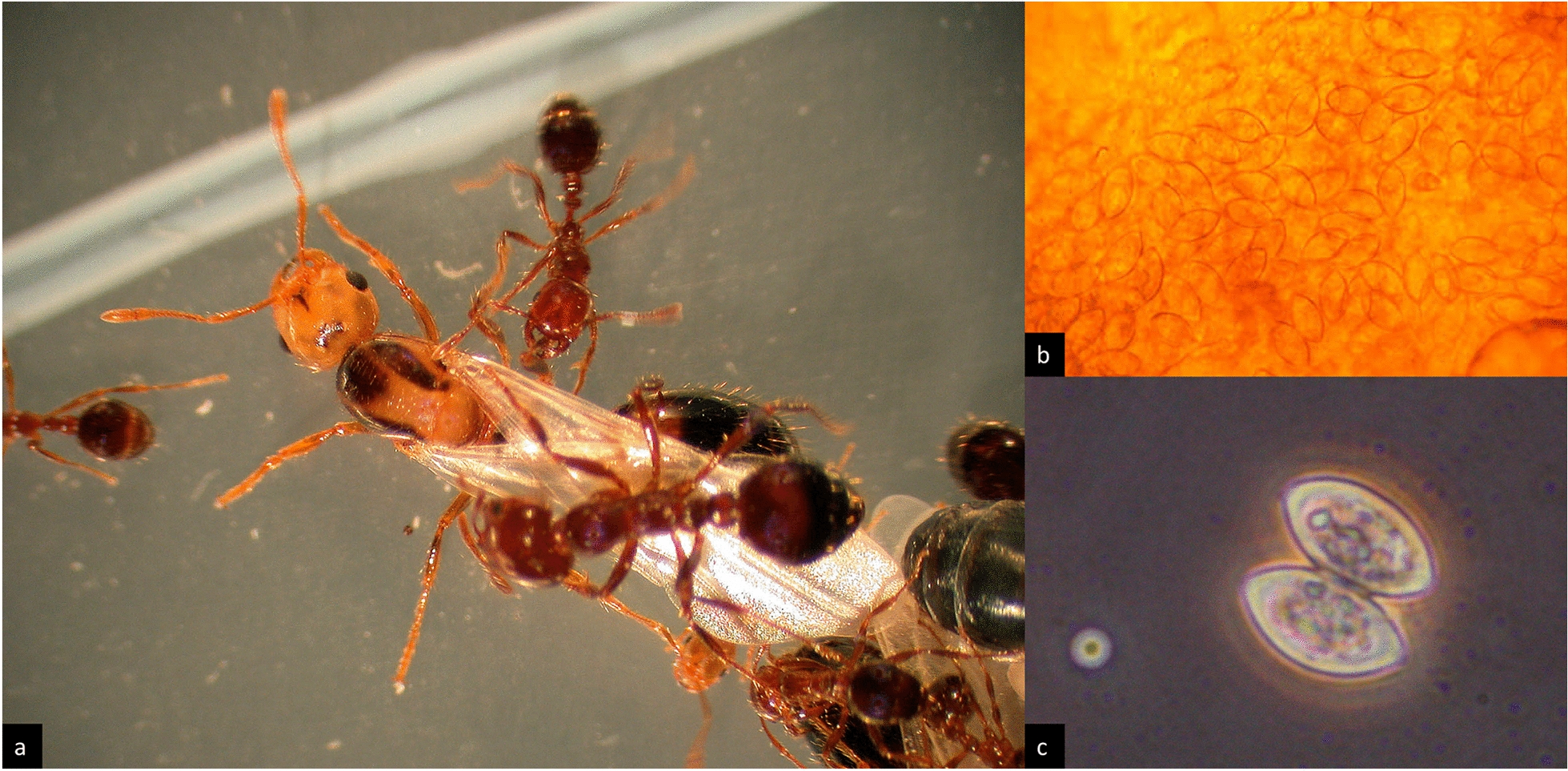
*Mattesia* sp. infecting *Solenopsis invicta*: **a** infected alate gyne of *S. invicta* surrounded by workers, note yellow head and thorax signifying “yellow-head disease”; **b** *Mattesia* sp. spores visible though cuticle of infected ant; **c** characteristic pair of lemon-shaped spores (photos: R. Pereira)

Parasitic unicellular organisms of the genus *Mattesia* (Order Neogregarinorida, Family Lipotrophidae), were first described from ants in 1979, upon identifying the infection in the fire ant *Solenopsis geminata* [124]. The parasite *Mattesia geminata* described in this study destructively invades oenocytes of the hypodermis and causes disruptions in the hosts’ preimaginal development, leading to melanization of the cuticle, reduced or discoloured compound eyes, and pupal death. Subsequent studies on multiple myrmicine host species from the USA, Canada, Brazil, and Europe (Table 2) yielded similar results, adding reduction of mandibular dentition to the characteristic syndrome and identifying preimaginal workers, gynes, and males as hosts [125–128]. A detailed account of the parasite’s complex life-cycle in hosts of the genus *Leptothorax* is provided in Kleespies et al. [127], showing characteristic tissue tropism: briefly, infective spores are ingested by host larvae; subsequent stages of the parasite develop extracellularly in the haemocoel, especially beneath the hypodermis and between lobes and cells of the fat body. In later stages, macronuclear merozoites invade the hypodermis and the fat body or settle extracellularly in the haemocoel. Upon maturity, two characteristic lemon-shaped spores (Fig. 5b, c) are developed in each gametocyst. In a laboratory setting, feeding infected pupae to ant larvae resulted in successful transmission of the parasite.

In contrast, the first described host, *S. geminata*, only presented with a limited range of infected tissues and parasite transmission in the lab was unsuccessful [124], leading to the assumption that it may not actually be a suitable host for *M. geminata* [127].

In the abovementioned cases, hosts were unable to attain imaginal maturity and died in the pupal stage. However, two cases of infection with *Mattesia* spp. of hitherto unresolved species identity are known to have produced aberrant adult ant phenotypes: the only published case of non-myrmicine hosts, namely workers of the Australian bull-ants *Myrmecia pilosula* and *M. rufinodis*, presented with a softer and lighter coloured exoskeleton, and increased mortality [129]. In workers and gynes of the invasive fire ant *Solenopsis invicta*, an infection with *Mattesia*-like spores resulted in the so-called “yellow-head disease” [130, 131]: host ants were recognizable by a yellow-orange discolouration of their head and parts of the thorax (Fig. 5a). Large workers were preferentially infected, though it is unknown whether the infection itself may alter imaginal size.

As some of the known hosts, e.g., *S. invicta* and *Monomorium pharaonis*, are known pest species of agricultural or medical importance, *M. geminata* has also received attention as a possible biological control agent [28, 127, 130].

### Fungi: *Myrmicinosporidium durum*

A recent surge of studies has dealt with investigating the phylogeny and effects of behaviourally manipulative fungal parasites in ants (e.g., *Ophiocordyceps* [29, 41], *Pandora* [132]). Despite the extensive literature on these so-called “zombie-fungi” and other fungal pathogens found in ant hosts (e.g., [133–135]), a review of hitherto published studies has yielded only one candidate putatively fitting the criteria of this publication: the enigmatic generalist fungal parasite *Myrmicinosporidium durum.*

First described by Karl Hölldobler from workers of *Solenopsis fugax*, the parasite was hypothesized to be of protozoan, perhaps haplosporidian origin [136–138]. Only in 1993 was it recognized as a fungal parasite and tentatively placed close to the order Chytridiomycetes [139], though newer studies place it within the Entomophthorales [140, 141]. Its true phylogenetic placement thus remains unresolved.

The parasite exhibits a remarkably generalist host range and wide distribution (see Table 1 in [142] and Table 2 for a complete list): cases have been reported from Central, southern and eastern Europe, the southern USA, the Galapagos Islands, and East Asia [141]. Ant hosts have been assigned to 40 species from three different subfamilies (Myrmicinae, Formicinae, Dolichoderinae) and may be queens, workers, soldiers, or males [136, 138, 141, 143–146]. While the details of its life-cycle remain elusive, previous authors have identified the ant host’s fourth larval instar as probable time of infection [139].

**Fig. 6:**
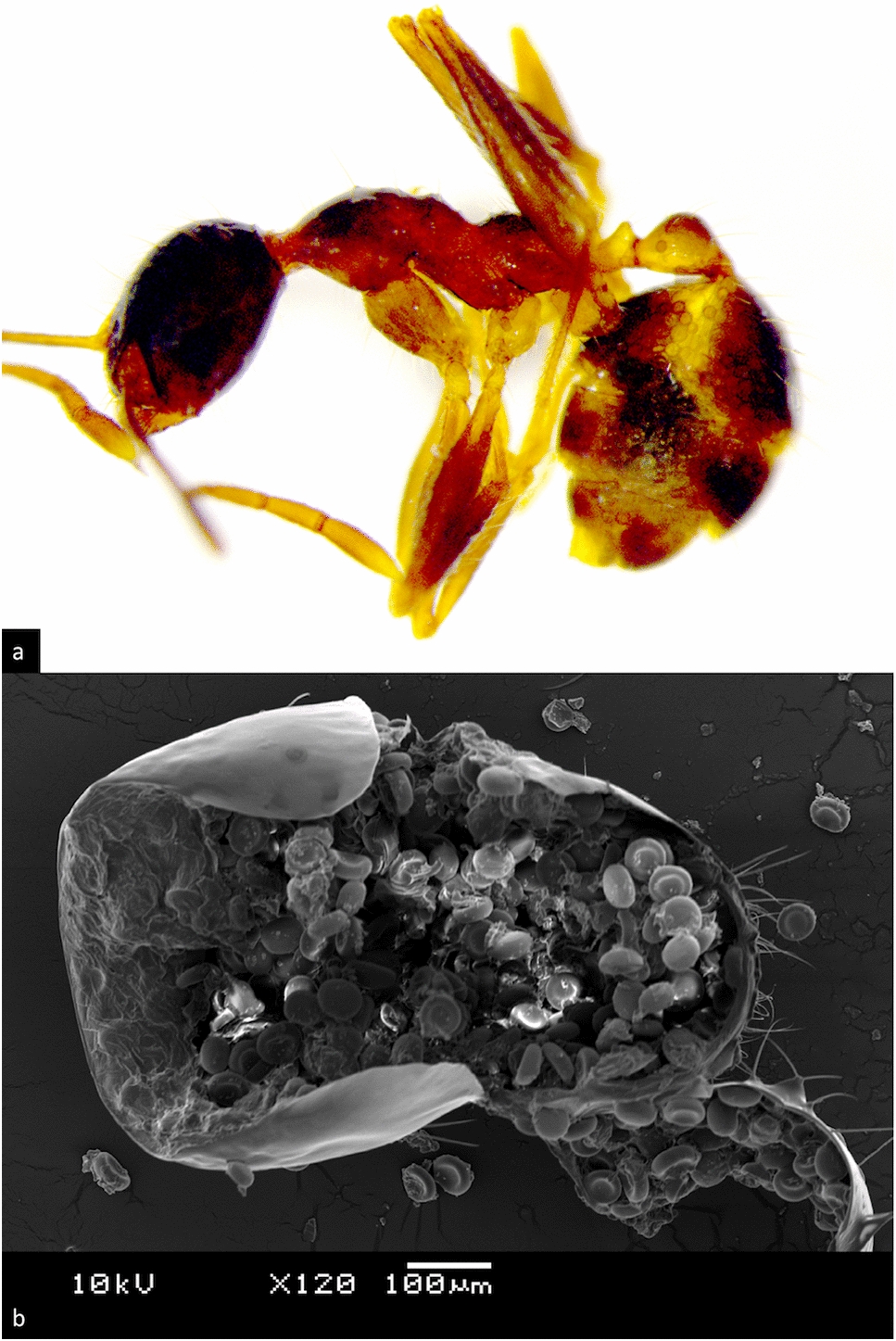
*Pheidole nodus* infected by *Myrmicinosporidium durum*: **a** minor worker, lateral view, note visible spores in gaster, petiole, mesosoma and coxae; **b** electron microscopic image of opened gaster, note lentil-shaped spores (photos: S. Hosoishi)

Infected ants with a light-coloured cuticle [145] are recognizable in the field by visible dark spores filling their gaster and—at later stages of infection—the entire body, even to the tips of the extremities (Fig. 6), though never the vital organs [136, 146, 147]. Spores are approximately 0.45 mm in diameter, lentil-shaped, and take on a characteristic bowl-like appearance when stored in alcohol, which inspired the German term “Näpfchenkrankheit” (lit. “little bowl disease”, Fig. 6b) [138]. Apart from the visible presence of the parasite, some authors have reported a distended, shiny, and darkened gaster in ant hosts [136, 138, 144, 147, 148], while others recorded no change in the appearance of infected ants [141, 149]. Increased mortality during hibernation or stress, depletion of fat reserves, and potential sterility of queens have been discussed as possible detrimental effects of the infection [136, 137, 146, 147], though other studies found no obvious negative influences and even reported a remarkable longevity of infected ants [133, 139].

This apparent lack of any strong detrimental effect on its diverse hosts has led to the hypothesis that *M. durum* may be a true generalist parasite with a long co-evolutionary history linking it to its hosts [133, 146, 148]. However, whether the observed morphological aberrations are truly the result of parasitic influence on host development and whether the occurrences reported from a wide range of different habitats and host taxa all represent the same parasitic species [141, 150] remains to be investigated in the course of further molecular and taxonomic studies.

### Viruses (?): “labial gland disease”

**Fig. 7:**
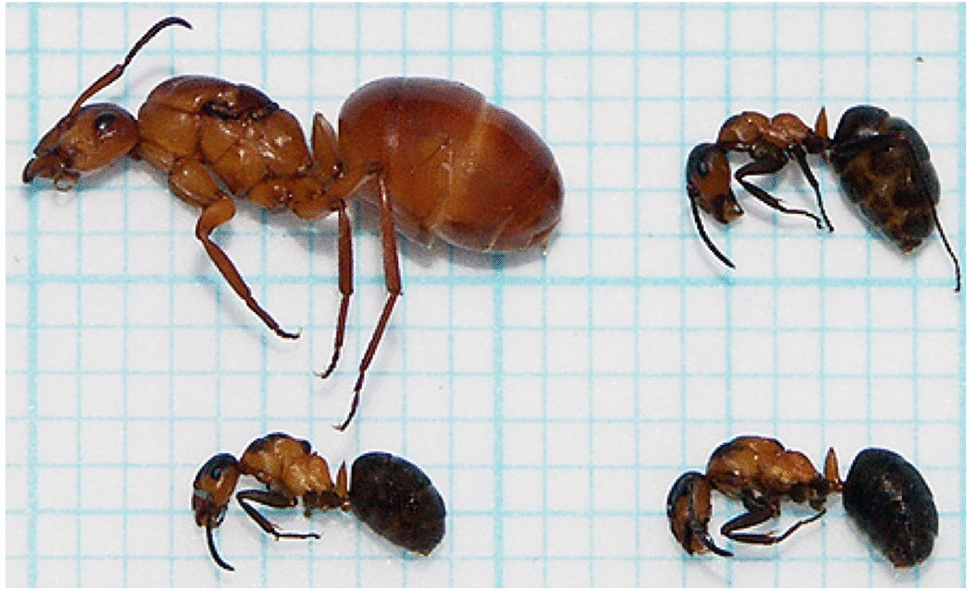
Labial gland disease in *Formica polyctena*; healthy queen (top left; colour bleached by storage in ethanol), healthy worker (top right), and two “pseudogyne” or “secretergate” specimens with labial gland disease (bottom, note humped mesosoma) (photo from [161])

This chapter is concluded by a hitherto unsolved mystery: in several species of formicine ants from Europe, the USA and Japan, the occurrence of individuals with characteristically malformed, enlarged mesosomas (Fig. 7) has been reported. Affected ants are known from at least ten species of *Formica*, as well as from two species, respectively, of *Camponotus* and *Prenolepis* [151–157].

The condition has been termed “labial gland disease”, as the swollen thorax is caused by the swelling of labial glands during the pupal phase [155]. Apart from the enlarged glands, the resulting workers (often termed “pseudogynes” sensu [151], or “secretergates” sensu [158]) are of normal size [153] or slightly smaller [159], exhibit a domed, gyne-like meso- and metanotum with variably defined sclerites, coupled with pale cuticle patches and increased pilosity on the affected regions, as well as increased mortality [153–156, 160]. Gynes (“secretogynes”) and males (“secretaners”) have also been reported to suffer from the condition. Secretogynes also show enlarged pronotums with lighter colouration and may have reduced wings and flight ability [152], but can mate normally and produce viable offspring with or without the disease [154].

As the causative agent remains unknown, the transmission of the disease can only be speculated about: the term “secretomorphs” for all affected individuals stems from the observed trophallaxis behaviour, whereby the sugary secretions of the enlarged labial glands are distributed to larvae and nestmates [158], which may transmit the disease to preimaginal stages. In *Formica fusca*, dead secretergates were found with holes bitten into their thorax and the labial glands removed, pointing to cannibalism as a potential mode of transmission [156]. Alternatively, accounts of secretergates developing from eggs of mated secretogynes without any observed feeding behaviour suggests a possible direct transmission from queen to offspring [154].

While earlier studies hypothesized the disease’s origin to be connected to the presence of myrmecophile beetles [151] or “erroneous” creation of intercastes by differential rearing conditions [152], the current—albeit unconfirmed—assumption is that of a viral pathogen [154]. If so, this would not be the first virus found to infect ants [22, 162]—a recently published review article reports 87 viruses found within 38 ant species across 15 viral families [162]—but hitherto the only one to cause such drastic and distinct morphological changes in its hosts.

## Discussion

### Parasites, development and evolution

**Fig. 8:**
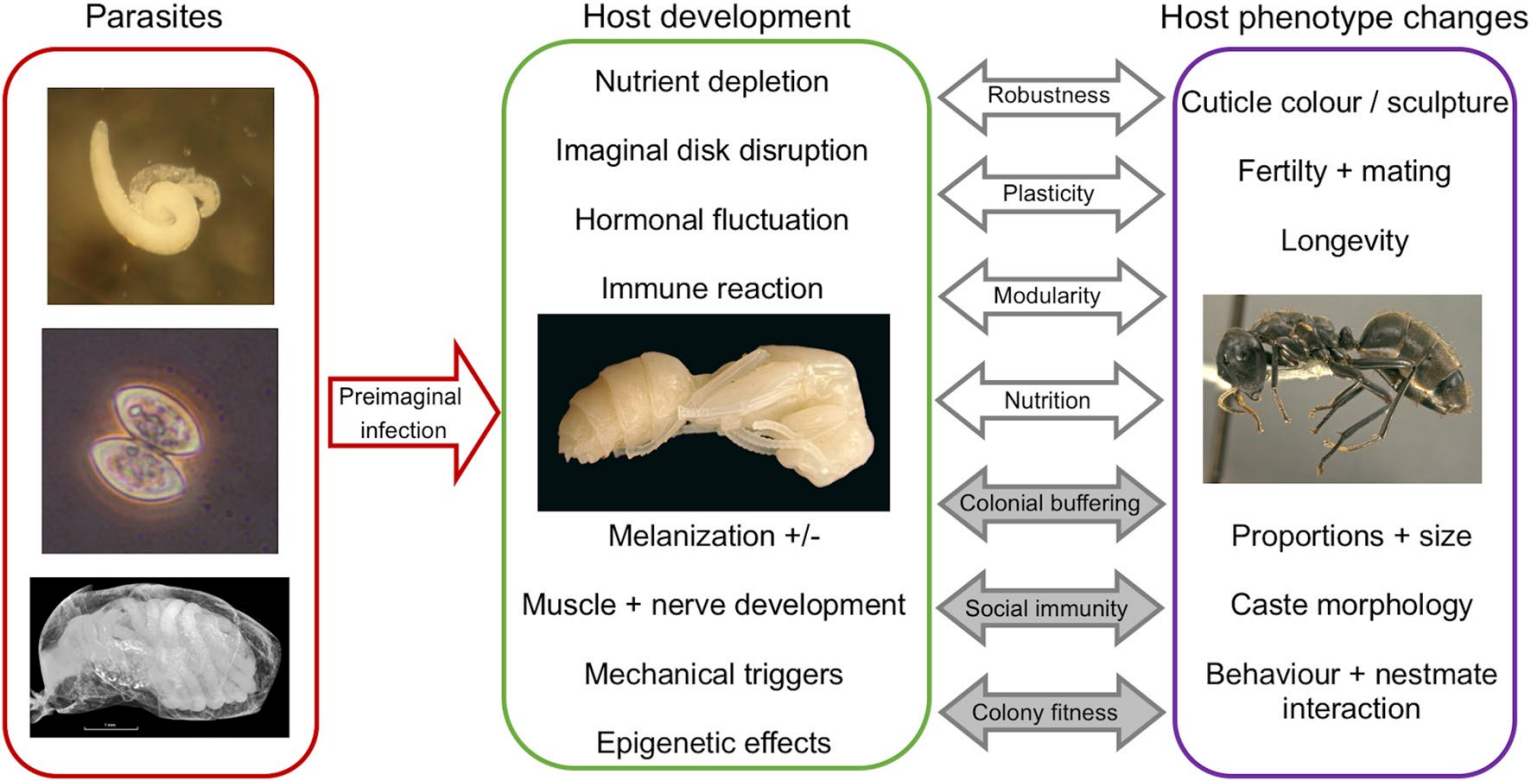
Summary of evolutionary and developmental factors mentioned in the literature surrounding parasite-induced phenotypes in ants; preimaginal parasitic infection (left box) may cause developmental perturbations (middle box), which are mediated by properties of individual ontogeny (clear arrows) as well as colony-level factors (grey arrows). This results in phenotypic changes to the host (right box), which may in turn mutually interact with further individual and colony-level evolutionary and developmental processes (photos: S.P. Yanoviak, R. Pereira, A. Laciny, [52])

Based on the diverse properties of the cases reviewed above, the immense variability and multidimensionality of parasitogenic phenotypes becomes apparent [34, 35] (Fig. 8). While some parasitic organisms elicit only subtle changes, such as altered colour of the cuticle, and do not seem to impede the host’s longevity or the fitness of the colony (e.g., *M. durum*), others may cause aberrations so severe they lead to utterly unrecognizable phenotypes (e.g., Mermithidae, Tetradonematidae) [70, 71, 73, 88] and even cause changes beyond the infected individual (e.g., *A. brevis*) [113, 122, 123].

While other arthropods may also be infected by parasites during development, the resulting effects are usually limited to changes in behaviour, parasitic castration, and depletion of fat reserves [31, 50, 163–166]. Except for a few accounts of parasite-induced intersex specimens in Mantidae [167], Culicidae [168], and Chironomidae [164], most cases present without any drastic morphological changes. Even within the social Hymenoptera, host species without pronounced caste polymorphism, such as bumblebees [169] and hornets [170] infected by mermithid nematodes, largely appear morphologically unchanged. The extremely altered phenotypes of parasitized ants thus seem to be linked to their particular developmental plasticity, especially in polymorphic species, where one genotype may give rise to multiple distinct phenotypes depending on environmental and colony-level conditions during rearing [30, 50, 67].

The mechanisms by which parasites trigger these changes have been hypothesized to involve chemical (e.g., [50, 91]), cellular [127], hormonal [69, 111], gene-regulatory [114], and nutritional [70, 118] disruptions during host development (Fig. 8), though—as highlighted throughout the cases presented—empirical data continue to be severely lacking for almost all host–parasite systems reviewed herein. The developmental effects underlying these changes are presumably as diverse as the phenotypes themselves and will have to be evaluated on a case-by-case basis in forthcoming research. However, certain parasite-induced syndromes (Table 1) are quite common and frequently present in a similar manner, even when comparing phylogenetically very distant host and parasite taxa. To highlight only a few examples: Davaineid cestodes, the fungus *M. durum*, and the unicellular parasite *Mattesia* sp. can all cause cuticular melanization in the host [99, 124, 136]; and both mermithid nematodes and dilepidid cestodes are known to cause reduced fertility and intercaste morphologies in infected gynes [40, 55]. The assumption that ontogenetic pathways necessary for typical ant development may be disrupted by different intruders in similar ways therefore appears valid, but requires further investigation in a controlled laboratory setting.

One of the few studies of gene expression in parasitized ants [114] found over 400 differentially expressed genes linked to parasite-induced changes at the individual or colony level in *T. nylanderi* parasitized by *A. brevis*. These differences in the transcriptome were linked to longevity, fertility, muscle growth and cuticular melanization—all traits which were subject to parasitic alteration in the studied species. Interestingly, healthy caste differentiation and the production of parasitogenic phenotypes may at least in part be governed by the same set of molecular mechanisms: e.g., *vitellogenin 3*, a gene associated with yolk protein production, fertility and caste differentiation [171, 172] was also found to be downregulated in ants infected by *A. brevis*, a parasite known to reduce fertility and produce intercaste phenotypes [114]. Vitellogenin depletion has also been connected to mermithid parasites causing parasitic castration in locusts [164]. Similarly, developmental disruption of the imaginal disks—crucial structures for ant caste differentiation [9, 10]—has been implicated in the production of parasitically altered phenotypes by the cestode *C. crateriformis* [118] and mermithized intersex specimens in Chironomidae [164].

In many ways, the study of parasite-induced phenotypes thus overlaps with ongoing investigations of caste evolution and development (e.g., [10, 11, 173–175]), with developmental timing and environmental factors playing key roles in both lines of research. In the cases reviewed herein, the presence of the parasite may take on the role of a developmental cue and shift the host onto a different ontogenetic trajectory, depending on the timing and severity of the infection [30, 32, 33]. As the pupa of holometabolous insects represent a closed system, previous authors have interpreted a parasite present during this developmental phase as a foreign tissue that may compete for resources with the host’s growing organs or influence the coordinated growth of imaginal structures [10, 86]. The resulting phenotypes often highlight not only the apparent plastic response of the host but also the remarkable robustness and modularity of ant development under environmental perturbation: previous studies [12, 53, 173, 174] have found ant body plans to be highly modular, i.e. while certain structures are tightly correlated through ontogeny and function, they are relatively independent of other such character clusters [176, 177]. Thus, parasite-induced changes to one set of body parts can result in drastically altered but still viable and largely functional phenotypes. This observation has led to the hypothesis that parasite-induced morphologies provide us with a window into the mechanisms underlying the evolutionary origins of novel castes and phenotypes in ants [11]: they may show us the limits of plasticity for a viable phenotype or serve as case studies to test currently competing EvoDevo models of caste origin [6, 8, 10–12, 175] (see also “Interpreting host phenotypes”).

In the examples provided above, the connections between parasitic infestation and host development—i.e. the “EcoDevo” [7]—are readily apparent, while the impact of the featured parasites on ant host evolution (“EcoEvo”) may be less obvious, especially as most hosts are non-reproductive workers. However, some parasites can have extremely high rates of infection, such as mermithid nematodes in *Lasius alienus* [63], *M. durum* in *Solenopsis fugax* [136], or dilepidid cestodes in *Temnothorax* spp. [118], infecting up to 40% of individuals in host colonies. As many parasites will affect caste composition, social behaviour, aggression, longevity or stress resistance [23, 112, 123], the cumulative effect of many infected workers and sexuals on the entire colony’s reproductive potential, survival, as well as inter- and intraspecific competition may certainly act as a selective pressures and shape evolutionary trajectories of both host and parasite [5, 23, 112, 122]. Notably, the significant effects parasites may have on their hosts’ life history and population ecology are the very basis of their frequently proposed use as mechanisms of biological pest control [19, 28, 127, 130, 170]. The general evolutionary processes underlying the establishment of host–parasite relationships through ecological fitting, as well as their shifts facilitated by environmental disturbances such as climate change, have been presented in detail elsewhere [178] and are certainly applicable to ant–parasite systems as well. Over an evolutionary timescale, non-heritable, environmentally induced characters—such as parasitogenic phenotypes—may even be fixed via genetic accommodation should they confer a fitness benefit to their hosts [4], though this currently remains a hypothetical possibility in need of further study.

### Interpreting host phenotypes

The vast diversity and variability of parasitogenic phenotypes provides not only fascinating insights but also important caveats for researchers. As outlined in “Methods”, one of the difficulties when working with such aberrant specimens is the correct interpretation of shifts in host size and caste identity, when morphological and developmental data on healthy phenotypes of the species are scarce—as is often the case. For example, an infected ant presenting with an intercaste morphology may be identified as an “enlarged” worker with “hypertrophied” queen-like characters by one researcher, but as a gyne exhibiting a “reduction” of all these structures by another—a drastic difference in the interpretation of the direction of parasitic effects on host development, as already lamented by Wheeler [50]. If, e.g., all mermithogenic morphologies in *Myrmica* come from queen-destined larvae, they thus result from reductions of queen-like characters (wings, thoracic sclerites, ocelli, ovaries), rather than the hypertrophy of these structures in workers [33]. This is not to say that parasitism generally cannot lead to hypertrophy: increased body size or relative elongation of appendages may certainly occur in cases where parasitic castration or reduction of the flight apparatus is correlated with compensatory growth in other structures (e.g., [31, 36, 52, 83, 179]; comp. [10, 86, 180]). But even in cases of parasite-induced increase of overall body size, the current state of knowledge about ant caste development makes it seem unlikely that an already worker- or soldier-destined larva could deviate from its developmental trajectory to express gyne-specific structures through parasitic influence [8–10, 181]. Interestingly, the existence of specimens exhibiting this kind of “gigantism” (gyne-like size, absent or weakly developed gyne-associated characters) seems to contradict a recently published model [175] of caste developmental evolution, which proposes a strong link between body size and gyne-like morphology (e.g., flight apparatus, ocelli).

A similar problem presents itself when constructing an explanatory narrative for the observed parasitic influences in an evolutionary context. As with parasite-induced behavioural changes [22], the proximate mechanisms of how parasitogenic morphologies arise remain largely unstudied. At the ultimate, adaptive level, many instances of parasite-induced changes to a host’s appearance and behaviour have been—and are still being—attributed to the parasite’s “extended phenotype”, i.e. the parasitic organism’s genome expressed through changes in the host [38, 39, 180]. However, this narrative of “adaptive manipulation” of host behaviour and appearance for the parasite’s benefit has been criticized as somewhat reductionist: parasite-induced alterations are often highly multidimensional and complex, comprising changes in appearance, ethology and physiology [23, 34, 35, 182]. Not all of these alterations confer increased fitness to the parasite—instead, they may also be adaptive responses of the host to infection or simple pathological reactions [26, 34, 35]. Drawing from the examples illustrated above, the syndrome summarized as “fruit mimicry” in *C. atratus* parasitized by *M. neotropicum* may indeed represent a complex of alterations (red gaster, weakened cuticle, slow movement) that benefit parasite survival and dispersal sensu Dawkins [38, 92]. In comparison, the effects of colonial buffering elicited by infection of *T. nylanderi* with *A. brevis* appear particularly beneficial for the survival of the host ants [112–114, 123]—from which the parasite may indirectly benefit as well. Contrastingly, there are currently no explanatory hypotheses linking the drastic morphological changes exhibited by ants harbouring mermithid nematodes to any kind of adaptive narrative beyond mere pathological reactions to metabolic disturbances during development. To disentangle these possibilities, differential transmission success linked to host phenotypes hypothesized to increase parasite fitness (e.g., [88, 89, 114]) may be analysed in field and laboratory settings. Further transcriptomic analyses to detect up- or downregulation of immunity-related genes in infected vs. uninfected ants [114, 123] are another important area of inquiry to better interpret the mechanisms underlying these changes.

To avoid any possibly misleading a priori assumptions, be it of “original” caste of the host or the underlying evolutionary narrative, a descriptive and comparative approach may be preferable in many cases, removing the need to assume causality or the direction of effect when it is still unknown. Thus, to provide a sound explanatory scenario for the observed phenomena, we as researchers should aim to evaluate host–parasite interactions on a case-by-case basis, considering what we currently know about the biological context of the respective system [35, 183].

### Outlook

Upon reviewing the literature on ant–parasite systems, one may conclude that while there is no shortage of studies describing single host–parasite associations and their phenotypic outcomes, empirical studies investigating the causal developmental mechanisms and processes are extremely rare. As many of the parasitic organisms discussed herein have multiple hosts and inhabit ants only at a certain stage of their life-cycle, the recreation of these natural experiments in a laboratory environment is often challenging. Therefore, the foundation of basic research necessary to build more sophisticated studies upon is still lacking, for many host–parasite systems, thus hampering further investigations.

The case of ant–parasitic mermithid nematodes (“Mermithidae” section) represents a good example of a system with an immensely rich body of case reports, but deficient in empirical investigations of developmental processes: apart from the vast diversity of aberrant host phenotypes, one of the most pressing obstacles to researching ant–mermithid systems is the unresolved taxonomy and diversity of the involved parasites [74]. Morphologically, mermithids are only reliably identifiable in their mature stage—and even then, only by expert nematologists [84, 85]—and their complex life-cycles are very difficult to recreate under controlled laboratory conditions [69]. Thus, mermithid nematodes often remain unidentified in a large portion of the literature.

Studies from the past decade focussing on DNA-barcoding of parasitic nematodes have yielded promising results for species identification [85, 184–186]. However, genetic sequences of ant–parasitic mermithids deposited in accessible databases are still extremely scarce, hampering identification and comparability even in cases where barcodes have been obtained [51, 74]. The same is true for methods of 3D imaging to assess the extent of internal and external changes in ant hosts (Fig. 1c): while microtomography has been successfully used to confirm and assess mermithid infection in ant hosts otherwise too fragile or valuable for dissection [52, 73, 187], more image data needs to be deposited accessibly to facilitate comparative research for future studies. In both cases, there is a dire necessity to establish comprehensive databases in order to facilitate future comparative research and link specific combinations of identified hosts and parasites to the phenotypic changes observed.

Upon completion of the necessary taxonomic and ecological groundwork to reliably identify a system of mermithid, ant host, and paratenic host viable in a laboratory setting, further empirical research could be conducted: previous studies, such as those on the topics of social immunity [26], caste determination [6, 10], or the *Temnothorax–Anomotaenia* system [114, 122, 123], offer numerous established laboratory protocols that may be applied to the ant–mermithid system as well. By thus assessing and comparing behaviour (e.g., nestmate interactions), longevity, imaginal disk development, or molecular analyses of transcriptomes and cuticular hydrocarbons of infected and uninfected individuals, we can hope to answer some of the many questions still remaining open to this day.

Future studies will necessitate novel, interdisciplinary research protocols and collaborative exchange of materials and knowledge across national and institutional borders. Ideally, such projects will combine fieldwork, taxonomy, morphometry, microtomographic imaging, statistical analysis, molecular methods, theoretical models, and critical assessments of historical and current literature. Basic research and the subsequent identification of suitable model organisms—for the ant–mermithid system and other host–parasite pairs—will facilitate further inquiries into open questions of genomic, hormonal, and behavioural consequences of parasitic infestation.

## Conclusions

Colonies of social insects such as ants interact not only with their conspecifics but also the ecological components of their environment—a multitude of symbiotic and pathogenic organisms among them. From miniscule fungal spores to worms as long as a human finger, ants are hosts to a plethora of parasites. The diverse ways in which these parasites may influence ant host morphology and behaviour provide fascinating examples of the interconnections between organisms and their environment. Parasites infecting ants in the preimaginal phase may act as triggers that disrupt normal ontogeny, thereby driving changes to morphology, gene expression and evolution. Assessing and comparing case studies across multiple host and parasite taxa allows us to explore beyond pure host–parasite associations and study responses to developmental stress, robustness and modularity of body plans or even the mechanisms governing the evolution of novel caste phenotypes. Despite the centuries-long research tradition surrounding ants and their parasites, most developmental processes underlying parasitogenic morphologies remain undiscovered, thus providing ample opportunity for forthcoming generations of scientists. As such, the identification and study of model host–parasite systems involving ants would greatly benefit the fields of EcoEvoDevo, myrmecology, and parasitology, as well as interdisciplinary collaborations among them. Such future theoretical and empirical studies will help us to further elucidate the complex roles parasites play in their hosts’ ecology, evolution and development.

## Data Availability

The datasets used and/or analysed during the current study are available from the author upon reasonable request.
